# Expanding the phenotype for the recurrent p.Ala391Glu variant in *FGFR3*: Beyond crouzon syndrome and acanthosis nigricans

**DOI:** 10.1002/mgg3.656

**Published:** 2019-04-23

**Authors:** Karen Rymer, Rita Shiang, Anting Hsiung, Arti Pandya, Tim Bigdeli, Bradley T. Webb, Jennifer Rhodes

**Affiliations:** ^1^ Department of Human and Molecular Genetics Virginia Commonwealth University School of Medicine Richmond Virginia; ^2^ Department of Psychiatry SUNY Downstate Medical Center Brooklyn New York; ^3^ Department of Psychiatry Virginia Commonwealth University School of Medicine Richmond Virginia; ^4^ Virginia Institute of Psychiatric and Behavioral Genetics, Virginia Commonwealth University School of Medicine Richmond Virginia; ^5^ Department of Surgery Virginia Commonwealth University School of Medicine Richmond Virginia; ^6^ Present address: Department of Pediatrics University of North Carolina North Carolina

**Keywords:** craniosynostosis, Crouzon, *FGFR3*, Pfeiffer

## Abstract

**Background:**

Craniosynostosis, or premature fusion of the skull sutures, is a group of disorders that can present in isolation (nonsyndromic) or be associated with other anomalies (syndromic). Delineation of syndromic craniosynostosis is confounded due to phenotypic overlap, variable expression as well as molecular heterogeneity. We report on an infant who presented at birth with multisuture synostosis, turribrachycephaly, midface hypoplasia, beaked nose, low set ears, a high palate and short squat appearing thumbs, and great toes without deviation. The additional MRI findings of choanal stenosis and a Chiari I malformation suggested a diagnosis of Pfeiffer syndrome. First tier molecular testing did not reveal a pathogenic variant.

**Methods:**

Whole exome sequencing on DNA samples from the proband and her unaffected parents was utilized to delineate the variant causative for the Pfeiffer syndrome diagnosis.

**Results:**

On whole exome sequencing, a de novo NM_000142.4:c.1428C>A missense variant causing a p.Ala391Glu amino acid change in *FGFR3* has been identified. The p.Ala391Glu change has been predominantly identified in patients with Crouzon syndrome with acanthosis nigricans.

**Conclusions:**

This finding illustrates the first reported case of a child with an overlap with Pfeiffer syndrome to have the p.Ala391Glu variant.

## 1 INTRODUCTION

Craniosynostosis (CS) or premature fusion of the skull sutures is a complex group of disorders that shows both clinical and molecular heterogeneity. It can lead to abnormal brain development and intellectual disability. Overall, CS presents in 1 in 2,500 births (Johnson & Wilkie, 2011) and either manifests in isolation (nonsyndromic) or with other anomalies (syndromic). Most syndromic cases involve complex synostosis often associated with limb anomalies, midface hypoplasia, and uncharacteristic facies (Derderian & Seaward, 2012). Three genes in the fibroblast growth factor receptor family (*FGFR1‐3*) have been shown to cause several forms of syndromic CS (Ciurea & Toader, 2009).

Classic Pfeiffer syndrome (type 1) (OMIM 101600) presents with bicoronal synostosis, broad thumbs and great toes, and variable levels of syndactyly. Pfeiffer syndrome types 2 and 3 are more severe and typically present with complex synostosis, midface hypoplasia, hearing loss, severe proptosis, short anterior cranial base, central nervous problems, gastrointestinal issues, and dental complications. There is the presence of a cloverleaf skull in type 2 (Jones, 2006; Robin et al., 1998).

Pathogenic variants in *FGFR1* (OMIM 136350) and *FGFR2* (OMIM 176943) are associated with Pfeiffer syndrome. In *FGFR1*, only the p.P252R variant has been associated with type 1 while pathogenic variants in *FGFR2* result in all three clinical subtypes. Pfeiffer syndrome pathogenic variants have not been identified in *FGFR3* (OMIM 134934). We report a patient with a clinical presentation that overlaps with Pfeiffer syndrome type 3 with a de novo* FGFR3* NM_000142.4:c.1428C>A change resulting in a missense p.Ala391Glu variant associated with Crouzon syndrome with acanthosis nigricans (CAN) (OMIM 612247). Our patient does not have the typical skin findings of acanthosis nigricans associated with this sequence variant even on follow‐up at age 9 (Arnaud‐López, Fragoso, Mantilla‐Capacho, & Barros‐Núñez, 2007; Meyers, Orlow, Munro, Przylepa, & Jabs, 1995). Ancanthosis nigracans normally occurs in childhood before puberty so the phenotype could still manifest.

## 2 MATERIAL AND METHODS

### 2.1 Ethical compliance

This study was approved by the IRB at Virginia Commonwealth University (VCU).

### 2.2 Whole exome sequencing (WES)

WES was performed on DNA samples from the proband (CS‐39) and her unaffected parents (CS‐54 and CS‐55) by Beckman Coulter Genomics (South Plainfield, NJ) using the Agilent SureSelect V5 (50Mb) capture kit (Santa Clara, CA) and paired‐end (2X100) sequencing via Illumina HiSeq. Mapping was performed using Burrows–Wheeler Alignment tool (v0.6.1‐r104) and human reference hg19. Library fragment sizes were estimated with Dindel (v1.01). Read map proportions were estimated using samtools (v1.18). Duplicate reads were marked using Picard (v1.86) and variant calling was performed using the GATK UnifiedGenotyper. Default parameters were used for all programs.

KGGSeq v1.0 was used to annotate, filter, and identify candidate recessive and de novo variants. Variants were (a) mapped to REFseq genes, (b) excluded if minor allele frequencies (MAF) were >0.01 in dbSNP138, 1,000 Genomes Project (April 2012 release), or NHLBI GO Exome Sequencing African Americans and European Americans datasets, and (c) kept if predicted to impact protein coding. KGGSeq pathway analysis was also performed to identify variants interacting with or within genes previously implicated in CS (Table S1). The Integrated Genome Viewer (IGV) (www.broadinstitute.org/igv/home) was used to confirm and visualize the presence of candidate variants and then confirmed using Sanger sequencing.

To identify possible variants affecting the skin phenotype, KGGSeq was used to identify deleterious variants in genes that were differentially expressed between human *FGFR3* or *PIK3CA* (OMIM 171834) mutant seborrheic keratosis (SK) and squamous cell carcinoma (SCC) (Hafner et al., 2007; Mandinova et al., 2009) as well as keratinocytes induced with mutant *FGFR3* versus wild type (Hafner et al., 2010), induction of *FOXN1*(OMIM 600838) (Janes, Ofstad, Campbell, Watt, & Prowse, 2004) and differences in nude (*FoxN1*) mouse keratinocytes versus wild type (Brissette, Li, Kamimura, Lee, & Dotto, 1996) filtered with a MAF of ≤0.05 (Table S2).

### 2.3 Sanger sequencing

Primers for PCR and Sanger Sequencing are found in Table S3. The PCR reactions contained 200 nmol dNTPs, 1X Phire Hot Start II buffer, 1.25 pmol/µl primers and 0.2 µl Phire Hot Start II polymerase (Thermo Scientific) in 25 µl volume. Cycling conditions were 98°C 1.5 min, (98°C 30 s, annealing temperature 30 s, 72°C 30 s) for 30 cycles, 72°C 7 min. PCR products were cleaned using ZymoResearch DNA Clean and Concentrator. Sanger sequencing was performed by the VCU Nucleic Acids Research Facility or Eurofins Genomics (Louisville, KY).

## 3 RESULTS

### 3.1 Clinical report

**Figure 1:**
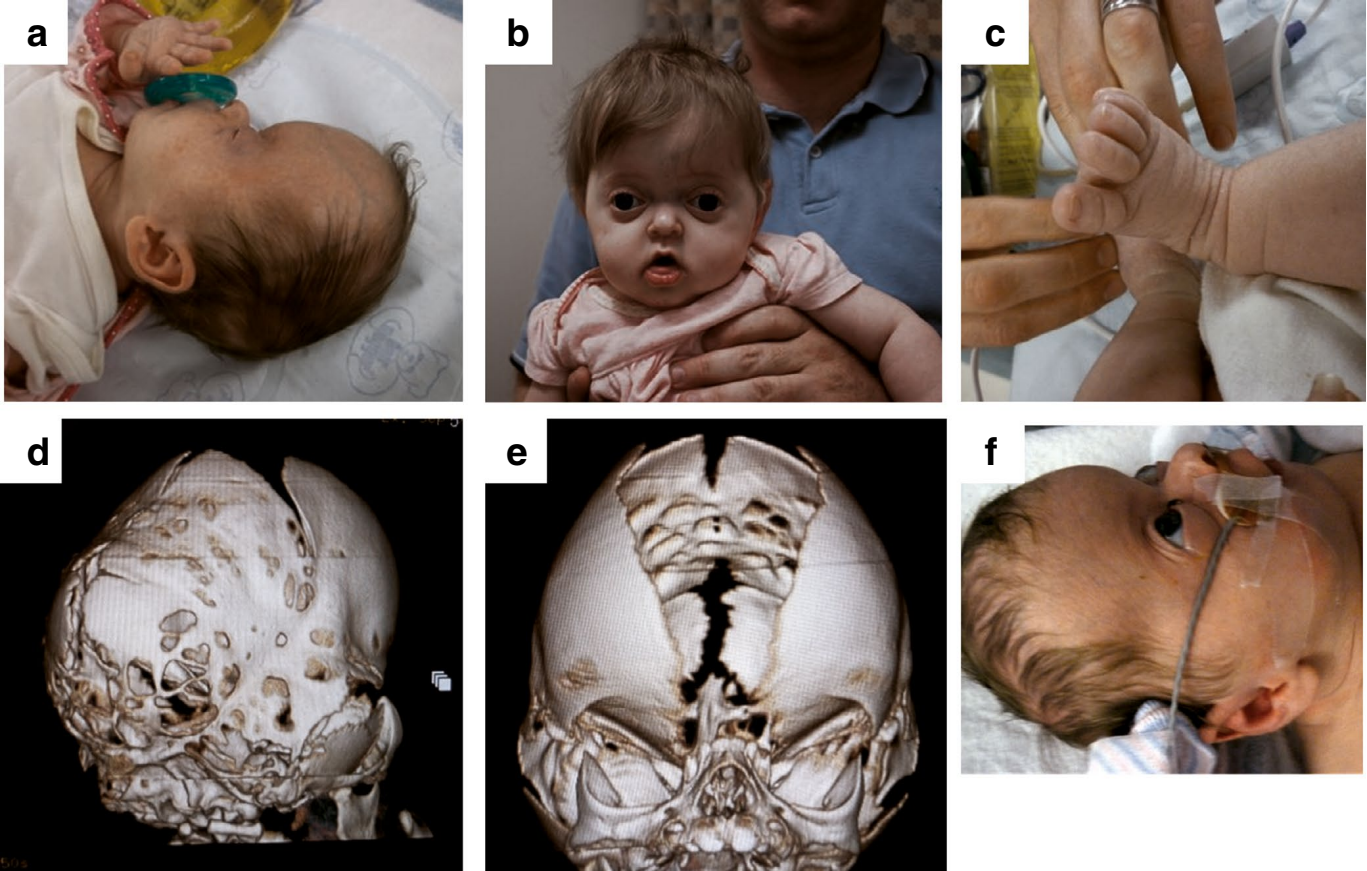
Clinical findings of CS‐39. (a) Severe turribrachycephaly and flattened lower occiput are evident. (b) Midface hypoplasia, beaked nose, and (c) broad toes are observed. (d, e) CT scans show lambdoid, metopic and bicoronal suture fusion. (f) Severe bilateral exorbitism and low set ears are observed

CS‐39 was ascertained by JR. She was delivered at home to a gravida 4 mother after an uncomplicated pregnancy and routine prenatal care after 8 weeks of pregnancy. Physical examination was notable for severe turribrachycephaly, coned superior occiput with flattened lower occiput, and moderate sub squamosal bulging (Figure 1a). Midface hypoplasia, beaked nose, narrow palate, low set ears and short squat appearing thumbs and great toes without deviation were noted (Figure 1b,c). CT scan showed a complex synostosis involving the metopic, bilateral coronal and lambdoid sutures (Figure 1d,e). MRI revealed choanal stenosis and a Chiari I malformation. The fontanelle was tense and bulging and the infant had noisy breathing. Tracheotomy was required to relieve obstructive sleep apnea. Additional findings include conductive hearing loss, strabismus, delayed gastric emptying, seizures, and kyphosis of her lower thoracic/upper lumbar spine. A clinical diagnosis of Pfeiffer syndrome type 3 was considered.

CS‐39's ophthalmology exam was notable for severe bilateral exorbitism, with full lid closure (Figure 1f) and down slanting palpebral fissures. Both pupils appeared normal. Dilated retinal exam indicated small to moderate hypoplasticity of the optic nerves, with the right optic nerve showing peri‐papillary pigmentation.

At 6 years of age, she has cognitive delay, is unable to feed herself and is nonverbal. She has moderate intellectual disability including sensory integration dysfunction and hyperactivity. A follow‐up MRI showed encephalomalacia in the right parietal and temporal lobes. Her parents and four siblings have normal clinical examinations.

### 3.2 Genetic findings

#### 3.2.1 Clinical testing results

Karyotype and high resolution chromosomal microarray along with pathogenic variant specific Sanger sequencing was performed on CS‐39. Given the Pfeiffer type 3 diagnosis, genetic tests were ordered for *TWIST1* (OMIM 601622) (entire gene), *FGFR1* p.Pro252Arg (rs121909627, NM_015850.3:c.749C>G), *FGFR3* p.Pro250Arg (rs4647924, NM_022965.3:c.749C>G), and *FGFR2* encompassing the IgIIIa and IgIIIc protein domains. No pathogenic sequence variants were identified.

#### 3.2.2 WES

WES was then performed on DNA samples of CS‐39 and her parents. On average, 98.7% of the reads were mapped to the reference genome and 68.4% of the reads were on target. A de novo NM_000142.4:c.1428C>A (previously c.C1172A) (rs28931615) change causing a p.Ala391Glu amino acid change in *FGFR3* was identified in CS‐39. This previously identified variant has been only associated in patients with CAN but very rarely is the skin phenotype not observed and none with digit anomalies (Roscioli et al., 2013). No additional predicted deleterious variants were identified in other known genes associated with CS. One additional de novo predicted deleterious variant was identified in *SPDYE4* (OMIM 617628) (Table 1). An additional 393 inherited rare predicted deleterious variants were identified (data not shown).

**Table 1:** Identified varients

Gene symbol	Gene description	Variant ID and reference sequence	Sequence change	MAF (ExAC_NFE)	Carriers of Alt Allele	Skin expression (mean RPKM)^a^	Functional consequence	Amino acid change	Deleterious predictions out of 21 tests^b^
de novo variants									
*FGFR3*	fibroblast growth factor receptor 3	rs28931615 NM_000142.4	c.1428C>A	ND	CS39	120.7	nonsyn SNV	p.Ala391Glu	10
*SPDYE4*	speedy/RINGO cell cycle regulator family member E4	NM_001128076.2	c.283C>T	0	CS39	0.005	stopgain	p.Arg95stop	NA
Downstream of FGFR3 Signaling									
*FBN2*	fibrillin 2	rs56168072 NM_001999.3	c.4312G>A	0.01	CS39, CS55	0.06	nonsyn SNV	p.Glu1438Lys	7
*NPHP4*	nephrocystin 4	rs200821373 NM_001291594.1	c.770A>T	0.0003	CS39, CS55	2.1	nonsyn SNV	p.His257Leu	12
*TP53BP1*	tumor protein p53 binding protein 1	rs45482998 NM_001141979.2	c.3092T>C	0.015	CS39, CS54	3.7	nonsyn SNV	p.Val1031Ala	8

Note

Alt: alternate allele; ExAC_NFE: ExAC_nonFinnish European; MAF: minor allele frequency; NA: not applicable; ND: no data; nonsyn: nonsynonymous; Ref: reference allele; RPMK: Reads Per Kilobase Milllion; SNV: single nucleotide variant.

a Fagerberg et al. (2014).

b SIFT, Polyphen2_HDIV or HVAR, LRT, MutationTaster, MuationAssessor, FATHMM, fathmm.MKL_coding, PROVEAN, VEST3, CADD, DANN, MetaSVM, MetaLR, integrated_fitCons, GERP, phyloP7way and 20way, phastCons2way and 20way, SiPhy_29way.

It is unknown if the lack of or possibly later‐onset of acanthosis nigricans in this individual is modified due to another variant. Since *FGFR3* is expressed in keratinocytes, a variant in *FOXN1*, a downstream gene, may affect the skin phenotype (Mandinova et al., 2009). No variants in the coding region of *FOXN1* was identified in our patient. A list of 77 genes (Table S2) was created by identifying transcripts differentially expressed when comparing activating variants in *FGFR3* or *FOXN1* to wild‐ type keratinocytes (Hafner et al., 2007,2010; Janes et al., 2004; Mandinova et al., 2009). These variants were filtered with a MAF ≤0.05 and predicted deleterious variants in three genes *FBN2* (OMIM 612570)*, NPHP4* (OMIM 607215), and *TP53BP1* (OMIM 605230) were identified (Table 1).

## 4 DISCUSSION

The classification of CS disorders has been through cataloguing features of individuals into distinct groups, although with significant overlap. Due to multiple suture involvement in CS‐39, Pfeiffer, Saethre‐Chotzen (OMIM 101400), or Crouzon (OMIM 123500) syndromes were considered. The findings of severe proptosis, short and broad thumbs and toes, choanal stenosis, cognitive delay, and seizures suggested a diagnosis of Pfeifer syndrome type 3.

The de novo FGFR3 p.Ala391Glu change identified was unexpected since pigmentary skin irregularities have not been observed in CS‐39 to date, and the craniofacial phenotype suggested Pfeiffer syndrome type 3. This variant has only been associated with CAN^6, 7^ and individuals without acanthosis nigricans are rare (<5%) (Roscioli et al., 2013). Crouzon syndrome is characterized by bicoronal synostosis, cleft lip or palate, midface hypoplasia, beaked nose, proptosis, and occasionally hearing loss with structurally normal hands, feet, and limbs. The CAN phenotype has been suggested to be a separate entity from Crouzon syndrome called Crouzonodermoskeletal syndrome (Arnaud‐López et al., 2007; Cohen, 1999). The p.Ala391Glu variant does explain the choanal stenosis as it is observed in 41%–43% of individuals with CAN (Arnaud‐López et al., 2007; Schweitzer et al., 2001) as well as Chiari I formation (23%) and hearing loss (14%). Vertebral malformations are present with a lower frequency in CAN and broad thumbs and big toes as well as developmental delay are very rare (Agochukwu, Solomon, & Muenke, 2014). No predicted deleterious variants were identified by WES in any other genes involved in CS to explain a more severe phenotype. The finding of the p.Ala391Glu variant in CS‐39 expands the phenotype associated with this specific variant and suggests further molecular heterogeneity with involvement of *FGFR3* with Pfeiffer syndrome. There is precedence of notable phenotypic variability reported for the p.Pro250Ala variant in *FGFR3* which can manifest as Muenke syndrome or nonsyndromic CS (Bellus et al., 1996).

A hypothesis for absence of skin changes in CS‐39 could be the presence of an additional sequence variant that could cause dysregulation downstream of FGFR3 signaling. Such a variant may be rare (MAF <0.01) but given that at least three individuals including CS‐39 with the p.Ala391Glu variant do not have skin findings (estimated <5%), a variant affecting this trait could be common (MAF >0.01) (Roscioli et al., 2013). The de novo variant in *SPDYE4* is not a candidate as its expression is restricted to the testes (Table 1) (Fagerberg et al., 2014). No coding variants were found in one candidate gene, *FOXNI*(Mandinova et al., 2009). Genes differentially expressed downstream of *FGFR3* or *FOXN1* in keratinocytes are also good candidates and predicted deleterious variants in three such genes *FBN2, NPHP4,* and *TP53BP1* were identified. These variants on their own may not manifest any phenotype as the variants in *FBN2* and *TB53BP1* are common but could modify the p.Ala391Glu phenotype. Sequencing of additional individuals with the p.Ala391Glu variants lacking or expressing delayed skin manifestations will need to be performed if variants modifying skin manifestations are to be elucidated.

The identification of this variant in CS‐39 contributes to the complexity in the understanding of the causes of syndromic CS disorders. As the underlying molecular etiology is identified in these cases, more phenotypical variation is associated with specific deleterious variants. The proband reported here has a very complex phenotype and not only lacked a symptom common (acanthosis nigricans) to p.Ala391Glu individuals but also manifested uncommon features (broad thumbs and big toes, hearing loss, developmental delay). This suggests that sequencing of a comprehensive panel of all major CS genes should be undertaken in circumstances of ambiguous clinical diagnosis.

## CONFLICT OF INTEREST

The authors declare no conflict of interest.
